# Trocar Puncture With a Sump Drain for Crohn's Disease With Intra-Abdominal Abscess: A 10-Year Retrospective Cohort Study

**DOI:** 10.3389/fsurg.2022.816245

**Published:** 2022-03-03

**Authors:** Juanhan Liu, Wenbin Gong, Peizhao Liu, Yangguang Li, Tao Zheng, Zhiwu Hong, Huajian Ren, Guosheng Gu, Gefei Wang, Xiuwen Wu, Yun Zhao, Jianan Ren

**Affiliations:** ^1^Department of General Surgery, Jinling Hospital, Medical School of Nanjing University, Nanjing, China; ^2^School of Medicine, Southeast University, Nanjing, China; ^3^Department of General Surgery, BenQ Medical Center, The Affiliated BenQ Hospital of Nanjing Medical University, Nanjing, China; ^4^Research Institute of General Surgery, Jinling Hospital, Nanjing, China

**Keywords:** Crohn's disease, intra-abdominal abscess, trocar, drainage, surgery

## Abstract

**Background:**

Traditional percutaneous catheter drainage (PCD) and surgical intervention could not always achieve satisfactory results for patients with Crohn's disease (CD) who have complications with intra-abdominal abscess. We proposed a trocar puncture with sump drainage for the treatment of CD with intra-abdominal abscess and compared it with the conventional PCD and surgical intervention.

**Methods:**

Crohn's disease patients with intra-abdominal abscess and admitted to our hospital from 2011 to 2020 were identified by reviewing the electronic medical records. We divided them into Trocar, PCD, and fecal diverting (FD) groups, according to the ways of treating an abscess. Outcomes, risk factors for abscess recurrence, and postoperative complications were compared among the three groups.

**Results:**

A total of 69 patients were included and they were divided into Trocar (*n* = 18), PCD (*n* = 29), and FD (*n* = 22) groups. Four patients in the PCD group were transferred to receive the FD surgery due to the failure of initial treatment. The incidence of abscess recurrence was significantly higher in the PCD (48%) and FD (50%) groups compared to the patients using the trocar puncture with the sump drain (Trocar group) (16.7%). There were 8 patients in Trocar, 22 in PCD, and 20 s in the FD group who received enterectomy. None of the patients in the Trocar had an ultimate stoma and the incidence of postoperative complications was statistically lower [0% (Trocar) vs. 31.8% (PCD) vs. 45% (FD), *P* < 0.05]. The way of initial treating of the abscess was significantly correlated with the abscess recurrence and postoperative complications.

**Conclusions:**

Trocar puncture with a sump drain had a lower incidence of abscess recurrence, abdominal adhesions, postdrainage, and postoperative complications compared to the conventional PCD or surgical intervention.

## Introduction

Crohn's disease (CD) is a chronic, idiopathic inflammatory gastrointestinal disease that has an increasing incidence worldwide (1). At least half of the patients require one or more surgical procedures during their lifetime due to the repeat flare-ups followed by clinical remission. The resultant extraluminal transit of gut flora can lead to abdominal infection and abscess formation or leak in more than 30% of patients with CD (2). The current guidelines of the European Crohn's and Colitis Organization (ECCO) recommend that active small bowel CD with a concomitant abdominal abscess should preferably be managed with antibiotics and percutaneous or surgical drainage followed by delayed resection, if necessary, and a defunctioning stoma to divert the fecal stream for the refractory CD was recommended (2).

In a recent meta-analysis, on comparing percutaneous catheter drainage (PCD) vs. surgery as the initial treatment for CD-related intra-abdominal abscess, it was found that the PCD) had no obvious advantage over surgery on ultimate permanent ostomy requirement, the occurrence of complications, or hospital stays. Additionally, the PCD resulted in a significant increase in abscess recurrence compared to surgery as the initial treatment (3). Given the fact that the PCD had no distinct benefits, currently, there is no better option for treating intra-abdominal abscess in patients with CD other than the PCD. Under such a situation, up to 70% of patients cannot avoid subsequent bowel resection. A recent prospective study for assessing the outcome of surgery after abscess healing had shown that surgery after successful nonoperative management of intra-abdominal abscess could provide good early and postoperative outcomes (4). But there is no general agreement as to the appropriate timing of the surgery.

Our previous study has preliminarily demonstrated that the trocar puncture with sump drain (5) had a lower incidence of postoperative complication and recurrence of abscess compared to the conventional Seldinger technique (6, 7). In our clinical 10-year practice, we reported cases using this trocar strategy successfully and satisfactorily.

This study aimed to explore the outcomes of the trocar with sump drain and we compared it with the conventional PCD and fecal diverting (FD) surgery in terms of complications after the initial treatment of abscess and definitive surgery, ultimate stoma creation, and the length of stay of patients with CD having intra-abdominal abscess. We also preliminarily explored the appropriate timing of the surgery.

## Materials and Methods

### Study Population and Definitions

We performed a retrospective review on all adult patients with CD between October 2011 and December 2020 in our institution. The diagnosis of CD was based on conventional clinical, radiological, endoscopic, and histopathological findings. All the patients included were ethnic Chinese. All the enrolled patients came from the same medical team of our hospital.

The inclusion criterion was a definite diagnosis of the abdominal infection on admission, which is defined as an abdominal abscess or leak by clinical signs (fever and abdominal pain), radiographic evidence (intra-abdominal free fluid, gas, and/or abscess on cross-sectional imaging or fistulography), and/or physical examination (abdominal mass, abdominal tenderness, rebound pain, and muscle tension). All the patients were treated with antibiotics once the infection was diagnosed and all the puncture drainage or surgical interventions were combined with the usage of antibiotics. Enteral nutrition was routinely used after drainage and surgery. After the puncture, a double lumen catheter was used to drain the abscess. The choice among using the trocar drainage, conventional PCD by a fine needle, or by FD surgery was made on a per-patient basis and left to the discretion of the surgeon. The FD surgery was mostly operated on emergency cases or those with relatively severe symptoms. The PCD or trocar is the most appropriate treatment for radiographically determined unilocular abscesses. The surgeon would comprehensively assess a patient's condition before making a decision on the available approach. Based on this, we selected the patients who utilized the trocar combined with the sump drain into the “Trocar” group, patients using a conventional percutaneous catheter into the “PCD” group, and patients who underwent laparotomy with FD surgery (temporary stoma) into the “FD” group.

Manual chart review, including clinician documentation, operative reports documented at the time of surgery, and imaging reports, was performed to confirm the accuracy of the CD and the abdominal infection diagnosis, surgical intervention, and relevant clinical data.

Patients treated by antibiotics only, turning to emergency bowel resection surgery, <6 months follow-up after surgery, <6 months follow-up after the initial treatment of abscess, or missing data were excluded.

### Procedures, Abscess Recurrence, Postdrainage or FD, and Postoperative Complications

**Figure 1:**
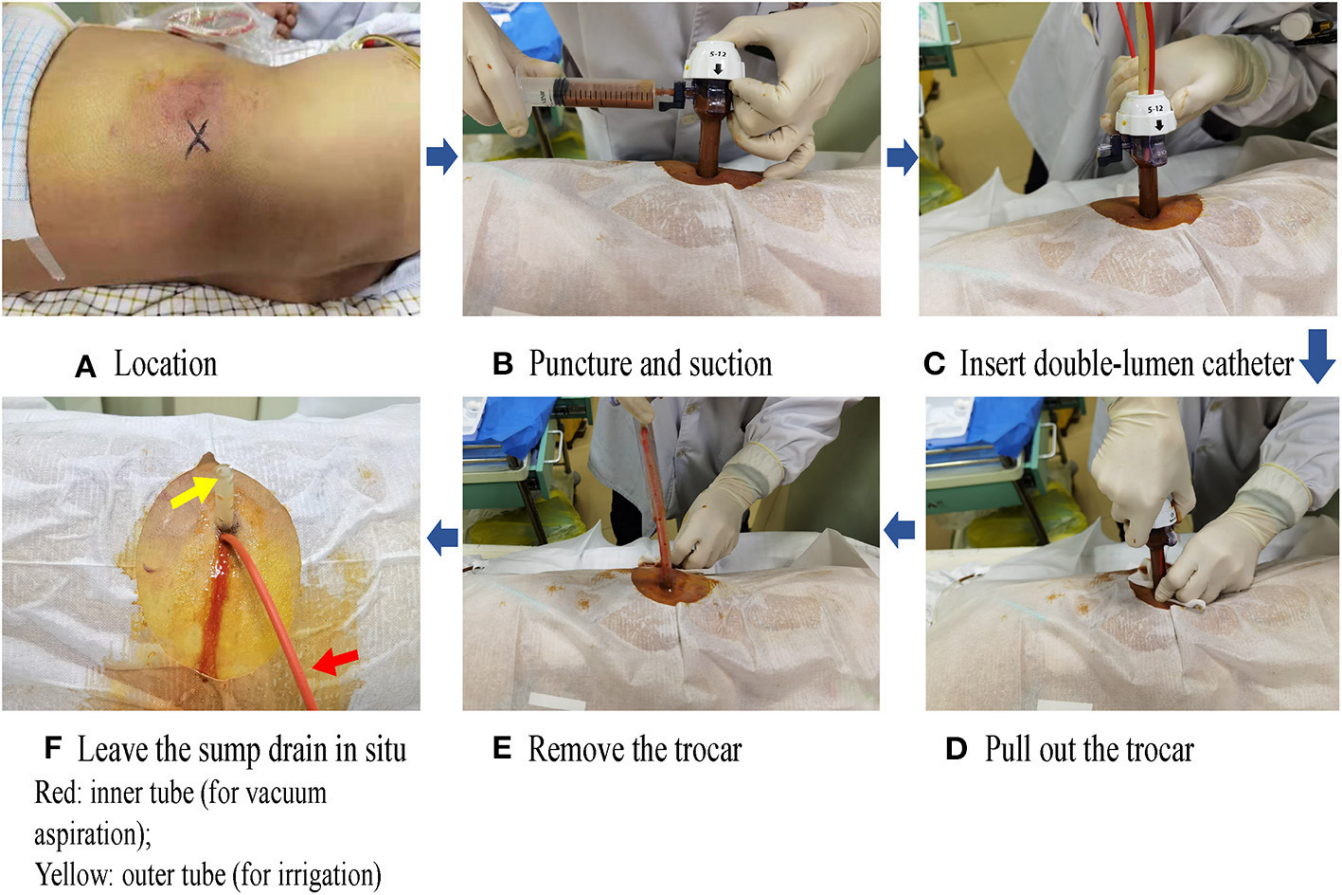
Procedure of trocar puncture with a sump drain. The actual figures and all tables have been uploaded as separate items. **(A)** Location, **(B)** Puncture and suction, **(C)** Insert double-lumen catheter, **(D)** Pull out the trocer, **(E)** Remove the trocer, **(F)** Leave the sump drain *in-situ* Red: Inner tube (for vaccum aspiration); Yellow: outer tube (for irrigation).

The description of the trocar technique is detailed in our previous publications (6–8). Briefly, the trocar puncture using a sump drain was defined as a procedure that used a 10-mm double-lumen irrigation-suction catheter (sump drain) replacing the conventional catheters to provide continuous irrigation and suction functions under negative pressure (Figure 1). This sump drain contains 2-channel drainage; the inner tube of the 2 channels is involved in vacuum aspiration (the red tube in the last picture of Figure 1), whereas the outer tube consists of several holes. The fluid inlet pipe is parallel to the outside casing and flushed with sterilized water. The whole device was placed in a 12-mm laparoscopic trocar puncture under the guidance of CT or ultrasound. After the trocar has been removed, the sump drain could be left *in situ*. The drainage tube could be removed after the clinical symptoms of the patient are resolved and after imaging that confirmed the disappearance of the abscess.

Abscess recurrence, length of stay, and complications were recorded during the follow-up period after the initial treatment of an abscess. Postoperative outcomes were evaluated by intra-operative findings when performing enterectomy, including abdominal adhesion, and outcomes after definitive surgery, including ultimate stoma creation and postoperative complications. The extent of resection was determined on the aspect of the diseased bowel and the patient's nutritional status. All postoperative events were assessed and graded by the Clavien-Dindo classification (9), and I–II grades were out of consideration in this study with no need for surgical, endoscopic, or radiological intervention. The duration of the follow-up period was defined as the interval between the discharge after the definitive surgery and the latest outpatient clinic visit or readmission.

Empiric antibiotics were initiated for our patients and it would be adjusted according to the antibiotic susceptibility testing. All the patients were treated with enteral nutrition initially through nasogastric or nasointestinal tubes during the hospitalization, if tolerable. For those who were intolerant to enteral nutrition, parenteral nutrition was given, and enteral nutrition would be established when their conditions improved clinically. Early use of immunomodulators would be encouraged based on the history of drug use after the patients' conditions became stable. The treatment of each patient after drainage or ostomy is heterogeneous, and the management process was to be adjusted by the surgeon according to the patient's condition, such as the course of antibiotic use and the nutritional support mode.

### Data Collection

Data collected included patient's baseline demographics [age, gender, current smoking status, and body mass index (BMI)], disease characteristics [disease duration, disease behavior, disease location, and perianal disease according to the Montreal Classification (10)], interval time between the healing of abscess and surgery, prior medication history within 3 months, and intestinal comorbidities. Intra-abdominal infection data included the size of abscess, abscess location, and the bacterial culture result of pus.

Complications were evaluated by recorded details as previously mentioned. All the baseline characters were included in univariate analysis and postdrainage abscess recurrence was considered as the variables related to postoperative complications.

### Statistical Analysis

Statistical analyses were performed with SPSS Statistics software (Version 26.0.0; IBM, Armonk, NY, USA). Quantitative variables were expressed as mean ± standard deviation or median with interquartile range, if necessary. Qualitative data were reported as frequency and percentage. Categorical variables were presented as proportions and compared with chi-square or Fisher's exact test, if necessary, while unpaired student's *t*-test or Mann–Whitney U test was used for comparing the quantitative variables. Univariate analysis was performed to identify the risk factors for the abscess recurrence and the occurrence of postoperative complications. Two-sided *P* ≤ 0.05 was considered significant.

### Ethics

This study was approved by the Medical Ethics Committee of Jinling Hospital, Medical School of Nanjing University.

## Results

### Demographics and Clinical Characteristics

**Figure 2:**
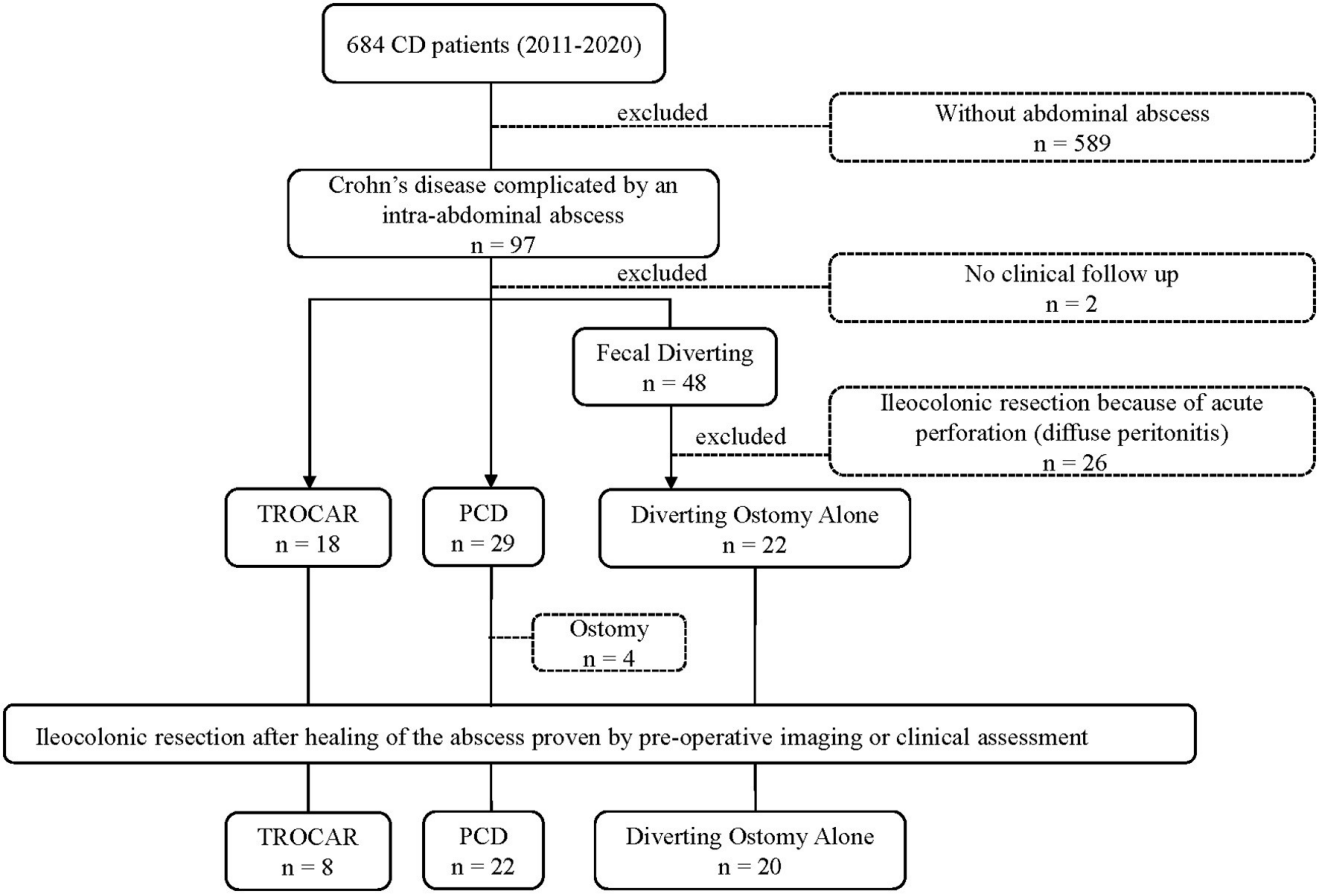
Flowchart of enrolled patients of all patients with Crohn's disease (CD) between October 2011 and December 2020 in the general surgery department of Jinling Hospital.

During October 1, 2011 and October 10, 2020, among the 684 adult patients with CD, 97 patients were detected as having intra-abdominal abscess, by imaging (Figure 2). Among them, 26 patients who underwent ileocolonic resection during laparotomy due to diffuse peritonitis and acute intestinal perforation and 2 having <6-month clinical follow-up were excluded. Among the remaining 69 patients, 18 patients were treated with trocar puncture, 29 used the conventional lower-diameter catheters to drain the pus, and 22 patients underwent FD with a temporary stoma. Overall, there were 69 patients fulfilling the inclusion criteria defining CD with intra-abdominal abscess. All of them were divided into three groups: Trocar, PCD, and FD according to the grouping method as mentioned earlier. Table 1 summarizes the characteristics of the treatment groups (Montreal classification, BMI, sex, duration of disease, smoking status, prior medication within 3 months, and intestinal comorbidities on admission), with no significant difference. The CT or fistulography had confirmed that all the enrolled patients had perforated disease. Before treatment, the mean size of abscess (maximum diameter) in the three groups were 6.35 ± 2.33 cm in the Trocar, 6.51 ± 1.89 cm in the PCD, and 6.77 ± 1.16 cm in the FD groups, respectively, with the value of *p* = 0.983 (Table 1). Multiple abscesses were detected in 1 (5.6%), 3 (12%), 1 (4.5%) in the TROCAR, PCD, and FD groups, respectively. Relatively, psoas, abdominal wall, and the intestinal loop were the predilection sites for abscesses in CD. Results of the bacterial culture in the pus showed that Escherichia coli was the overwhelming presence in the TROCAR (5 patients), PCD (6 patients), and FD (6 patients) groups, with no significant difference.

**Table 1:** Baseline characteristics of patients with abdominal abscess.

	**All patients** **(***N*** = 69)**	**TROCAR** **(***N*** = 18)**	**PCD** **(***N*** = 29)**	**FD** **(***N*** = 22)**	***P*** **value**
Age (year, mean ± SD)	33.14 ± 10.57	33.44 ± 8.746	32.64 ± 9.891	33.45 ± 12.894	0.957
>40	6 (6.74%)	3 (7.14%)	3 (6.98%)	2 (9.09%)	
≤ 40	79 (93.26%)	39 (92.86%)	40 (93.02%)	20 (90.91%)	
BMI (kg/m^2^)	18.09 ± 3.13	18.06 ± 3.20	17.76 ± 3.39	18.17 ± 2.78	0.898
Sex					0.651
Male	42 (64.6%)	10 (55.6%)	17 (68.0%)	15 (68.2%)	
Female	23 (35.4%)	8 (44.4%)	8 (32.0%)	7 (31.8%)	
Duration of disease (month, mean ± SD)	43.32 ± 36.51	32.94 ± 35.03	53.40 ± 38.73	40.36 ± 33.65	0.175
Active smoker	3 (4.6%)	1 (5.6%)	1 (4.0%)	1 (4.5%)	0.971
Disease location (Montreal classification)					0.405
L1 (ileal)	24 (36.9%)	6 (33.3%)	7 (28.0%)	11 (50.0%)	
L2 (colonic)	16 (24.6%)	5 (27.8%)	7 (28.0%)	4 (18.2%)	
L3 (ileocolonic)	25 (38.5%)	7 (38.9%)	11 (44.0%)	7 (31.8)	
L4 (upper digestive tract)	0 (0%)	0 (0%)	0 (0%)	0 (0%)	
Prior medication within 3 months					0.335
None, *n* (%)	21 (32.30)	5 (27.80)	10 (40.00)	6 (27.30)	
5-aminosalicylates, *n* (%)	17 (26.0)	5 (27.80)	7 (28.00)	5 (22.70)	
Immunosuppressants, *n* (%)	13 (20.00)	5 (27.80)	2 (8.00)	6 (27.30)	
Corticosteroids, *n* (%)	6 (9.20)	3 (16.70)	1 (4.00)	2 (9.10)	
Infliximab, *n* (%)	8 (12.30)	0 (0.00)	5 (20.00%)	3 (13.60%)	
Intestinal comorbidities					0.643
Fistula	34 (452.30)	10 (55.60)	15 (60.00)	9 (40.90)	
Perforation	19 (29.20)	5 (27.80)	5 (20.00)	9 (40.90)	
Obstruction	9 (13.80)	3 (16.70)	3 (12.00)	3 (13.60)	
Others^a^	3 (4.60)	0 (0.00)	2 (8.00)	1 (4.50)	
Abscess size (max diameter [cm]) (mean ± SD)	6.55 ± 1.97	6.35 ± 2.33	6.51 ± 1.89	6.77 ± 1.16	0.983
Abscess location, *n* (%)					0.792
Psoas muscle	18 (27.69)	7 (38.90)	7 (28.00)	4 (18.18)	
Abdominal wall	19 (29.23)	6 (33.30)	8 (32.00)	5 (22.12)	
Interloop	14 (21.54)	2 (11.10)	3 (12.00)	9 (40.90)	
pelvic	9 (13.85)	2 (11.10)	4 (16.00)	3 (13.60)	
Multiple abscesses	5 (7.69)	1 (5.60)	3 (12.00)	1 (4.50)	
Bacterial culture					0.69
Not available	30 (46.15)	8 (44.40)	11 (37.94)	11 (50.00)	
Escherichia coli	18 (27.69)	5 (27.80)	7 (24.13)	6 (27.30)	
Klebsiella pneumoniae	7 (10.77)	3 (16.70)	4 (13.79)	0 (0.00)	
Enterococcus faecium	6 (9.23)	1 (5.60)	3 (10.34)	2 (9.10)	
Proteus mirabilis	6 (9.23)	1 (5.60)	2 (6.90)	2 (9.10)	
Acidobacter froudi	3 (4.61)	0 (0.00)	2 (6.90)	1 (4.50)	

a *Perforation Repair, Endoscopic polyp extraction, Exploratory Laparotomy, anemia, pancreatitis, pneumonia*.

### Outcomes After the Initial Management of Abscess

All the enrolled patients were followed up for more than 6 months (Table 2). During this period, the rate of readmission in FD (90%) was significantly higher than in the Trocar (61.11%) and the PCD (75.9%) groups, including but not limited to the reason of performing ileocolonic resection and managing recurrent abscess, intestinal obstruction, intestinal fistula, or perforation. Four of 29 (13.7%) in the PCD group could not get a satisfactory result and turned to a FD surgery finally. Total (*p* = 0.026) postdrainage or FD (*p* = 0.003) and the length of stay was lower in the Trocar (10.44 ± 4.35 days, 7.72 ± 3.83 days) and the PCD (10.60 ± 11.05 days, 6.52 ± 4.79 days) than in the FD group (17.50 ± 10.69 days, 11.00 ± 4.48 days), which had no significant difference in the first two groups. Three in Trocar (16.7%) had recurrent abscess, notably less than PCD (48%, *p* = 0.033) or FD (50%, *p* = 0.028). None of the patients who used the trocar drainage had postdrainage complications during the follow-up period. Four (16%) patients who had used the traditional lower-diameter catheters drainage and 6 (30%) patients who had undergone enterostomy had unhealed or not disappeared intestinal fistulas. Twelve (54.55%, *p* < 0.001) patients in the FD got stoma-related complications during the follow-up time, including 4 parastomal fistulas, 4 stoma stenoses, 2 stoma prolapses, and 2 parastomal hernias. Cost on the infection control was significantly lower in the trocar drainage and in the conventional PCD groups than performing a FD surgery (*p* < 0.001).

**Table 2:** Outcomes of the initial management of abscess.

	**TROCAR**	**PCD**	**FD**	***P*** **value**
Follow-up time, month (s) (mean ± SD)	14.13 ± 3.56	13.16 ± 2.74	11.03 ± 9.01	0.989
Readmission, *n* (%)	11 (61.11)	22 (75.9)	20 (90.9)	0.084
Length of stay, day(s) (mean ± SD)	10.44 ± 4.35 ^a^	10.60 ± 11.05	17.50 ± 10.69	0.026*
Hospital stay after infection source control, day(s) (mean ± SD)	7.72 ± 3.83^b^	6.52 ± 4.79^c^	11.00 ± 4.48	0.003*
No definitive surgery, *n* (%)	10 (55.56)	3 (12.00)	2 (9.09)	0.000*
Unhealed intestinal fistula, *n* (%)	0 (0.00)	4 (16.00)	6 (30.00)	0.041*
Abscess recurrence, *n* (%)	3 (16.70) ^d, e^	12 (48.00)	11 (50.00)	0.059
Complications after drainage and laparotomy, *n* (%)	0	0	12 (54.55)	0.000*
Parastomal fistula	0	0	4 (18.18)	
Stoma stenosis	0	0	4 (18.18)	
Stoma prolapse	0	0	2 (9.09)	
Parastomal hernia	0	0	2 (9.09)	
Hospitalization expenses				
Surgery (¥), mean ± SD	33,371.1 ± 12,641.5	39,055.7 ± 34,106.3	82,955.4 ± 22,241.9	0.000*

a *p = 1.000, TROCAR vs. PCD*.

b p = 0.044*, *TORCAR vs. FD. p = 0.578, TROCAR vs. PCD*.

c p = 0.002*, *PCD vs. FD*.

d p = 0.033*, *TROCAR vs. PCD*.

e p = 0.028*, *TROCAR vs. FD*.

*P ≤ 0.05 is in bold and asterisked*.

### Comparison of the Surgical Procedure, Intraoperative Findings, and Postoperative Outcome of Subsequent Ileocolonic Resection

There were 4 in Trocar, 22 in PCD, and 20 patients in FD who received ileocolonic resection after the healing of the abscess. More than 6 months of postoperative follow-up was aimed to observe the postoperative outcomes of different types of drainage. Choice of surgical approach was statistically different among the three groups (*p* = 0.035). The laparoscopic method was performed in 4 (50%) patients using the trocar drainage, 3 (13.64%) using lower-diameter catheters, and 2 (10%) who underwent enterostomy (Table 3).

**Table 3:** Comparison of surgical procedure, intraoperative findings, and postoperative outcomes after surgery.

	**TROCAR**	**PCD**	**FD**	***P*** **value**
Number	8	22	20	
Interval time between source control and definitive surgery, month (s), (median, (range interquartile))	3, (0.25, 6.0) ^a^	3, (1.0, 5.0)	9, (5.25, 25.0)	0.001*
Surgical approach				
Laparoscope, *n* (%)	4 (50.00)	3 (13.64)	2 (10.00)	0.035*
Open surgery, *n* (%)	4 (50.00)	19 (86.36)	18 (90.00)	
Abdominal adhesion, *n* (%)	4 (50.00)	12 (54.50)	15 (75.00)	0.011*
Postoperative complications ^a^, *n* (%)	0 (0.00%) ^b, c^	7 (31.8) ^d^	9 (45)	0.070
Cholestasis	0	0	2	
Intestinal fistula	0	2	5^e^	
Intra-Abdominal infection	0	2	3	
Wound infection	0	1	2	
Intestinal Obstruction	0	2	2	
Others	0	0	2^e^	
Ultimate stoma creation, *n* (%)	0 (0.00)	13 (52.00)	7 (31.82)	0.001*
Death, *n* (%)	0	0	0	-

a *All complications were graded as III-IV by the Clavien-Dindo classification*.

b *p = 0.084, TORCAR vs. PCD, Fisher exact test*.

c p = 0.021*, TROCAR vs. FD, Fisher exact test.

d *p = 0.288, PCD vs. FD, Fisher exact test*.

e *3 in 5 patients also occurred intra-abdominal infection; Acute respiratory distress syndrome happened on 2 of 5 patients with fistula*.

*P ≤ 0.05 is in bold and asterisked*.

The timing of the surgery after successful drainage is controversial. Table 3 shows that the interval time between the healing of the abscess and the ileocolonic resection was significantly shorter in the Trocar and PCD groups; The interval time between the healing of abscess and definitive surgery in patients using the trocar was 3 (0.25, 6.0) months and 3 (1.0, 5.0) months in PCD, whereas, it was 9 (5.25,25.0) months in the FD group (*p* = 0.001). The incidence of intraoperative diagnosis of abdominal adhesion was significantly lower in the Trocar group (*n* = 4, 50%) and 12 in the PCD group (*n* = 12, 54.5%) with or without the clinical manifestation of intestinal obstruction.

Patients using the trocar drainage performed one-stage intestinal anastomosis (*n* = 4) after enterectomy and no postoperative complications occurred during the follow-up period. Ultimate stoma creation rates among the three groups were significantly different. Patients using the trocar with no need to reverse the stoma ended with no ultimate stoma creation while 13 in the PCD (52%) and 7 in the FD (31.82%) groups had the stoma formed when discharged. After an intestine enterectomy, 7 (45%) patients in the PCD had postoperative complications requiring surgical intervention, including 2 intestinal fistulas, 2 intra-abdominal infections, 1 wound infection, and 2 intestinal obstructions. In the FD group, 9 (45%) postoperative complications occurred, comprising 2 cholestasis, 3 intra-abdominal infections combined with intestinal fistulas, 2 wound infections and 2 intestinal obstructions, and 2 acute respiratory distress syndromes combined with intestinal fistulas. Both of these two groups both had an obvious higher incidence of postoperative complications than the Trocar group, with a statistical difference between the Trocar and the FD group (p = 0.021) (Table 3).

### Risk Factors of Abscess Recurrence and Postoperative Complications

Univariate analysis was performed to identify the risk factors of abscess recurrence after the initial treatment and postoperative complications after intestinal resection. Table 4 shows that the recurrence of abscess was significantly related to disease behavior (*p* = 0.045) and different ways of abscess treatment (*p* = 0.038). The type of drainage also had a strong correlation with the incidence of postenterostomy complications (*p* = 0.014). Moreover, the abscess recurred after the initial treatment was associated with the postoperative complications (*p* = 0.013).

**Table 4:** Risk factors of abscess recurrence after initial treatment of abscess and postoperative complications.

**Variable**	***P*** **value** **(abscess recurrence)**	***P*** **value** **(post-operative complications)**
Sex	0.533	0.238
BMI	0.143	0.438
Age (Montreal classification)	0.203	0.432
Disease behavior (Montreal classification)	0.045*	0.147
Disease location (Montreal classification)	0.456	0.367
Duration of disease	0.437	0.295
Active smoker	0.342	0.293
Prior medication within 3 months	0.483	0.945
Intestinal comorbidities	0.649	0.203
Multiple abscess	0.558	0.264
Different ways of drainage	0.038*	0.014*
Abscess recurrence	-	0.013*
Timing of surgery	-	0.073
Surgical approach	-	0.194

*P ≤ 0.05 is in bold and asterisked*.

## Discussion

For CD complicated with intra-abdominal infection, effective source control in the intra-abdominal infections needs to be performed before the definitive surgery (11). In this regard, PCD is advised as the primary treatment for well-defined unilocular abscesses when accessible by interventional radiology and with reported successful drainage rates of 74–100%(2, 12–15). Under ultrasound and CT guidance, the PCD is a safe procedure with a low complication rate. For those patients with complex lesions or the presence of refractory CD, such as complicated with intra-abdominal infection or intestinal fistulas beyond the control of medical treatment, the latest guideline of ECCO has mentioned that a defunctioning ileostomy to divert the fecal stream would be needed which allows for remission and facilitates perioperative optimization (2). Especially for those emergency patients, ostomy (surgery without bowel resection) was necessary. The meta-analysis mentioned that a comparison of the PCD and surgery on the patients with CD who had intra-abdominal abscess (3) showed that the PCD did not demonstrate apparent advantages over surgery in relation to the complications and the length of stay, and the mean size of abscess in this research was 5.6–11.1 cm, which is similar to that in this study (6.55 ± 1.97 cm). In the recent research analyzing the modality of treatment for intra-abdominal abscesses in 3,296 hospitalized patients with CD, 39% were treated with antibiotics alone, 29% with percutaneous drainage, and 32% with surgery; under the condition with no difference on abscess characteristics, the data had shown a significantly higher risk of abscess recurrence following the PCD (OR: 6.544, 95% CI: 1.783–24.010, *P*: 0.005) than the surgery (16). Currently, there is a paucity of data addressing the efficacy of these therapies in comparison with surgical management. Most patients included in this study had a long medical history and the mean duration of disease was 43.32 ± 36 months. This implied that most of them had experienced a long natural history of the disease, which also leads to its heterogeneous nature, and the patients could not get satisfactory treatment or cured simply by antibiotics therapy or PCD, and ended with surgical intervention, such as temporary stoma or diseased bowel excision. But aggressive strategy (temporary ileostomy) may result in a greater likelihood of subsequent definitive surgery or even a permanent stoma.

Traditional percutaneous drainage in the treatment of intra-abdominal abscess in CD has been evaluated in other studies with an eventual surgical therapy rate of 20% to 70% (16). In our study, 13.7% of patients with PCD had to receive FD surgery due to failed infection source control. And even with successful PCD, two-thirds of patients cannot guarantee one-staged surgery or no subsequent surgery (17, 18). Our data also showed that 52% of patients in the PCD group cannot avoid a two-staged surgery, which is lower than the former research (70.7%) (3) possibly due to the limited number of included patients in this study. In the meantime, all the patients using the trocar performed successful primary anastomoses, and no drainage failure happened after the initial treatment of an intra-abdominal abscess.

Although trocar cannot avoid subsequent enterectomy just like the PCD or FD, intestinal resection remains as the standard approach after a complete resolution of the abscess to avoid the recurrence of septic complications and facilitates control of the disease in the recent studies (4).

The timing of surgery after successful drainage is still controversial. A previous study (14) was in favor of selective surgery considering that the diseased bowel left intact will be a source for future abscess recurrence. In one study, 6 of 7 patients who did not have subsequent enterectomy had a severe recurrence within 3 years (19). However, early surgery may also result in disease relapse, ultimate stoma creation, and higher re-operation rates. Another research showed that 48 patients were performed surgery at a median of 43 days (range 8–220 days) after percutaneous abscess drainage. Twenty-three percent of these patients required a stoma (20). A recent multicenter, international cohort study comprising 335 patients with CD patients having PD followed by surgery showed a short-waiting interval, <2 weeks after PD, was associated with a high incidence of abscess recurrence (OR 0.59 (95%c.i. 0.36–0.96), *P* = 0.042) (21). Mean interval time of our study between the surgery and drainage was about 3 months in TROCAR and PCD groups. All patients receiving diverting surgery had waited more than 1 year (median time was 9 months). The results showed that the interval time had no significant different effect on the incidence of postoperative complications, which may be due to the relatively long interval than the other studies. The kind of effect an early surgery will have on disease relapse needs more prospective studies and a longer follow-up time. Meanwhile, a precise definition of the appropriate interval time or the timing of surgery is also necessary. In addition, the PCD and surgery caused more postoperative complications and a higher rate of ultimate stoma creation than patients using trocar drainage.

Puncture was done based on two main categories: the Seldinger technique and the trocar technique (22). The Seldinger technique used a thin needle to puncture, which is easily blocked by the necrotic tissue, blood coagulates, or by the purulent fluid in the abscess. Our study showed that a puncture with a 12-mm laparoscopic trocar which had a larger diameter and draining by double-lumen catheter could effectively avoid undrainable thick pus and persistent intra-abdominal fluid ending with a low incidence of abdominal adhesion, which will decide whether a surgeon could perform laparoscopic surgery to reduce intraoperative blood loss and its related complications (23). In this regard, a trocar with a double-lumen catheter had a distinct advantage over the conventional lower-diameter catheters drainage.

Risk factors for complications after the first ileocecal resection for CD have been introduced before (4, 24–26). Poor nutritional status (27–29), intra-abdominal abscess discovered during surgery (30), preoperative steroids (31), the use of biologicals for more than 3 months, and the recurrent clinical episode of CD were mostly focused. We also investigated the risk factors for abscess recurrence and postoperative outcomes. Disease behavior did have some relation with abscess recurrence (*p* = 0.045), which was correlated with postoperative complications (*p* = 0.013). We found that the way of abscess treatment was associated with abscess recurrence and postoperative complications. Notably, we did not find any correlation between the usage of steroid and the development of abscess.

At present, the optimal treatment approach to CD complicated with an abscess that could be successfully treated with medical management remains controversial. A conservative approach (antibiotics with or without percutaneous drainage) is frequently taken before performing a surgery. Intestinal resection and anastomosis after the healing of abscess have been increasingly used. Traditional PCD with incomplete drainage increases the difficulty of surgery and the risk of disease recurrence. Our institution has used trocar combined with a double-lumen catheter draining for more than 10 years, and the data showed that trocar had a great advantage in shortening the hospitalization time, hospitalization cost, and reducing abscess recurrence, as well as the incidence of abdominal adhesion. There is a paucity of data addressing the effect of surgery on the different ways of abscess treatment. Long-term follow-up after surgery also reveals that trocar combined with a double-lumen catheter draining was a good choice to effectively reduce the complications after surgery and ultimate stoma creation.

Currently, the trocar puncture has not been widely used in clinical practice, and the number of patients who are able to be included is limited in this study, which might be due to selection bias. We hope this study could bring an optional drainage way for patients with CD patients who had complications with intra-abdominal abscess. This is a retrospective single-center study, with nonuniformly distributed enrolled patients, which brings a possible selection bias. More prospective researches in the future need to be conducted.

## Conclusion

Trocar puncture with sump drain would be a better option for the management of intra-abdominal abscess in patients with CD. It would result in shorter hospital time, lower incidence of abscess recurrence, and better outcomes after the management of abscess and subsequent definitive surgery.

## Data Availability Statement

The original contributions presented in the study are included in the article/supplementary material, further inquiries can be directed to the corresponding author.

## Ethics Statement

The studies involving human participants were reviewed and approved by Medical Ethics Committee of Jinling Hospital, Medical School of Nanjing University. Written informed consent for participation was not required for this study in accordance with the national legislation and the institutional requirements.

## Author Contributions

JL designed the work, contributed to data collection, analysis, and manuscript writing. WG wrote and revised the manuscript and contributed to data analysis. PL and YL enrolled patient selection, contributed to data collection, analysis, and revised the manuscript. TZ participated in the operation, designed the work and revised the manuscript. ZH, HR, GG, and GW participated in the operation and management of patients, contributed to data collection and analysis, and revised the manuscript. XW designed the work, wrote and revised the manuscript, and checked data. YZ designed the work, revised the manuscript, and checked data. JR contributed to implementation of surgery or other intervention, designed the work, and revised the manuscript. All authors contributed to the article and approved the submitted version.

## Funding

This study was financially supported by the 333 High-Level Talents Training Project of Jiangsu Province (BRA2019011).

## Conflict of Interest

The authors declare that the research was conducted in the absence of any commercial or financial relationships that could be construed as a potential conflict of interest.

## Publisher's Note

All claims expressed in this article are solely those of the authors and do not necessarily represent those of their affiliated organizations, or those of the publisher, the editors and the reviewers. Any product that may be evaluated in this article, or claim that may be made by its manufacturer, is not guaranteed or endorsed by the publisher.
